# Skiagraphy in the Diagnosis of Urinary Calculi

**Published:** 1901-01-26

**Authors:** 


[1] Lancet, Jan. 19.

### SKIAGRAPHY IN THE DIAGNOSIS OF URINARY CALCULI.

In the diagnosis of urinary calculi great assistance may be obtained by the use of the Roentgen rays, whether the calculi be situated in the kidney, the ureter, or the bladder, and in many cases this may be the only method by which a fixed stone may be discoverable. Dr. Mansell Moullin[1] says that unless there is some unusual difficulty, such as an abnormally stout abdomen or an extreme lumbo-dorsal curvature, displacing and burying the kidney, the Roentgen rays can be relied upon to give definite and accurate information, not only as to the existence of a calculus, but as to its size, its exact position, and, what is even more important, as to whether there are other calculi present, either in the same or in some other part of the urinary tract. The best results are of course obtained with oxalate of lime calculi, as these absorb the rays to a much greater degree than others; but even concretions of phosphate can be made to throw a distinct shadow, unless they are so minute as to be of no practical importance. Thus a negative result can now be relied upon as much as a positive one. Even in cases where there is no doubt as to the existence of a stone the rays may be of great service, especially in the case of renal calculi. One may know that there is a stone, but may be quite in the dark as to its size, its position in the kidney, and as to whether there may not be other stones hidden away deep in one of the calices. A single examination with the X-rays may clear up all these points. Under ordinary circumstances the rays are not required for the diagnosis of vesical calculi. But when occurring in combination with enlargement of the prostate even a vesical calculus is very easily overlooked. It may not give rise to characteristic symptoms, and it is very easy to miss it with the sound. It often happens in these cases that the only indication of its presence is the obstinate persistence of cystitis, which resists all ordinary treatment; and Dr. Mansell Moullin is strongly of opinion that whenever this is a prominent feature in association with enlargement of the prostate a special examination for calculus should be made without delay. Persistence of cystitis in the face of treatment thoroughly carried out points to some lasting cause which is much more often a calculus than is usually imagined. In some of these cases of enlarged prostate neither the sound nor the cystoscope can be trusted. Sometimes the latter cannot even be introduced. But with the Roentgen rays all is made easy, without pain or inconvenience, without introducing anything into the bladder, and without running the risk of an anæsthetic.

